# Gadolinium toxicity: mechanisms, clinical manifestations, and nanoparticle role

**DOI:** 10.1007/s00204-025-04124-x

**Published:** 2025-07-03

**Authors:** Jose L. Domingo, Richard C. Semelka

**Affiliations:** 1 Laboratory of Toxicology and Environmental Health, School of Medicine, Universitat Rovira i Virgili, Sant Llorens 21, Catalonia, 43201 Reus, Spain; 2 Consulting. PLLC, Chapel Hill, NC USA

**Keywords:** Gadolinium-based contrast agents (GBCA), Gadolinium toxicity, Nephrogenic systemic fibrosis (NSF), Gadolinium deposition, Nanoparticles, Chelate stability

## Abstract

Gadolinium-based contrast agents (GBCAs), essential for MRI, are facing renewed scrutiny due to gadolinium (Gd) retention and emerging toxicity profiles. While the link between less stable agents and Nephrogenic Systemic Fibrosis (NSF) in renal impairment is established, gadolinium (Gd) deposition is also observed in the brain, bone, and skin across all GBCA classes, even in patients with normal renal function. This finding has raised concerns and led to a concept of Gadolinium Deposition Disease (GDD). The present review synthesizes current evidence on clinical manifestations and underlying mechanisms. It highlights pathways beyond traditional transmetallation, particularly endogenous nanoparticle formation as a key mechanism for Gd release and retention, potentially challenging the stability assumptions for even macrocyclic agents. Structural factors (linear/macrocyclic; ionic/non-ionic) and stability parameters (thermodynamic log K; kinetic kobs) influencing risk are evaluated alongside regulatory responses. GBCAs should be viewed not as inert diagnostics but as agents with complex, cumulative biological interactions. Future research should focus on developing non-gadolinium alternatives, validating biomarkers for early detection of Gd retention, and conducting controlled trials on chelation therapy efficacy. Clinicians must balance the diagnostic benefits of GBCAs with potential long-term risks, ensuring informed patient consent and judicious use. Innovative approaches, such as Gd-grafted nanodiamonds with high relaxivity and enhanced safety via polyvinylpyrrolidone (PVP) coating, may offer alternatives to traditional GBCAs by reducing toxicity risks*.* Manganese-based contrast agents, such as Mn-PyC3A, show promise as safer alternatives due to efficient renal and hepatobiliary elimination, even in renal impairment, as demonstrated in rat models.

## Introduction

In contemporary medical diagnostics, contrast-enhanced magnetic resonance imaging (MRI) serves as a crucial modality, providing superior soft tissue visualization and functional data (Aguet et al. 2022; Naijar 2024). Central to this technique are gadolinium-based contrast agents (GBCAs). These agents employ the paramagnetic characteristics of the gadolinium ion (Gd^3^⁺) to shorten T1 relaxation times, thereby enhancing image contrast (Caravan et al. 1999; Kim et al. 2018; Do et al. 2020). This enhancement capability is often essential for diagnosing and tracking a broad spectrum of conditions, such as cancer, inflammatory processes, and neurological issues, yielding information not attainable with other imaging methods or non-contrast MRI. Their substantial contribution to diagnostic precision and patient care management solidifies their essential place in modern medicine, despite ongoing safety discussions (Starekova et al. 2024). Since the US FDA first approved a GBCA in 1988, millions of doses have been utilized worldwide. Initially, GBCAs presented a robust safety record, with adverse event rates documented between 0.001% and 0.01% (Murphy et al. 1996; Prince et al. 2008).

GBCAs feature a trivalent gadolinium ion (Gd^3^⁺) enclosed within an organic ligand chelate. Chelation is vital, because the unbound Gd^3^⁺ ion is highly toxic. Its ionic radius is similar to calcium’s, allowing interference with critical calcium-dependent biological processes (Ersoy and Rybicki 2007). The ligand isolates Gd^3^⁺, reducing toxicity and enabling rapid elimination via the kidneys (Tweedle et al. 1988). The perception of GBCA safety was dramatically altered in 2006 with the identification of nephrogenic systemic fibrosis (NSF). This severe fibrotic illness showed a strong connection to GBCA administration in individuals with profound renal impairment (Cowper et al. 2000; Grobner 2006; Marckmann et al. 2006, 2008; Perazella 2009). Implementing screening practices and favoring more stable GBCAs significantly reduced NSF incidence (Wang et al. 2011). Nonetheless, a new safety question surfaced in 2014 with reports of increasing signal hyperintensity on non-contrast T1-weighted MRI scans in specific brain regions after multiple doses, predominantly involving linear GBCAs. Significantly, this was observed even in individuals with normal renal function (Errante et al. 2014; Kanda et al. 2014; McDonald et al. 2015; Murata et al. 2016). Later research confirmed Gd presence in various tissues such as the brain, bone, and skin among people previously given GBCAs (Radbruch et al. 2015; Guo et al. 2018). This phenomenon of Gd deposition, occurring to varying degrees, is linked with all GBCA categories (Port et al. 2008; Kanal and Tweedle 2015; Radbruch et al. 2015; Coimbra et al. 2024). A more contentious subject concerns patients reporting lasting symptoms post-GBCA exposure, giving rise to the concept of “Gadolinium Deposition Disease” (GDD) (Burke et al. 2016; Semelka et al. 2016a). Davies et al. (2022) provided a comprehensive summary detailing the contemporary understanding of Gd pharmacokinetics, toxicity pathways, and the range of clinical issues, emphasizing chelate stability and the generally better safety record of macrocyclic vs. linear agents.

Mechanistic investigations have challenged established notions of Gd toxicity. While transmetallation (the displacement of Gd^3^⁺ by endogenous metals) was considered the principal mechanism for Gd release from less stable chelates (Idée et al. 2006), subsequent findings suggested more complex pathways (Taupitz et al. 2013; Gianolio et al. 2017). Transmetallation occurs when Gd^3^⁺ is displaced from its chelating ligand by metals naturally occurring in the body. Intriguingly, emerging data indicate that the in vivo generation of Gd-containing nanoparticles could be a significant factor in Gd retention and toxicity (Coimbra et al. 2024). Endogenous molecules like oxalate might initiate this process within specific biological microenvironments (Taupitz et al. 2013; Henderson et al. 2025).

Considering this context, the present review intends to synthesize the current knowledge base on GBCA-associated toxicity. It concentrates on clinical manifestations, deposition patterns, and the evolving understanding of underlying mechanisms. By integrating recent findings, particularly regarding nanoparticle formation, this review presents a detailed view of the risk–benefit profile of these agents while also identifying critical areas needing further research. GBCAs are classified by key characteristics that dictate their stability and safety. Understanding these categories is essential for evaluating the variable toxicity risks among different agents. In addition, novel approaches like Gd-grafted nanodiamonds, which offer high relaxivity and enhanced safety through polyvinylpyrrolidone (PVP) coating, are explored as potential alternatives to traditional GBCAs (Panich et al. 2016, 2019, 2021; Chizhikova et al. 2024).

## Search strategy

An extensive search of the literature was executed using Scopus, PubMed, and Embase, spanning publications from the late 1980 s to April 2025. The search involved free-text terms and MeSH terms where applicable, utilizing keywords, such as: “gadolinium,” “gadolinium-based contrast agents,” “GBCA,” “MRI contrast,” “toxicity,” “adverse effects,” “safety,” “nephrogenic systemic fibrosis” (NSF), “Gadolinium Deposition Disease” (GDD), “transmetallation,” “nanoparticles,” “kidney disease,” “chelating agents,” and “chelation therapy.” Boolean operators (AND, OR) were employed to refine queries. Manual review of bibliographies from significant studies, reviews, and guidelines supplemented the electronic search. Regulatory documents from agencies such as the US Food and Drug Administration (US FDA) and the European Medicines Agency (EMA) were also consulted. Materials included comprised original preclinical, clinical, and in vitro research; systematic reviews; meta-analyses; case reports/series (especially for NSF/GDD); authoritative reviews; clinical guidelines; and regulatory statements, confined to English-language publications. Titles and abstracts were initially screened, followed by full-text assessment based on inclusion criteria. Preference was given to studies significantly enhancing comprehension of the pathophysiology, clinical aspects, risks, diagnosis, treatment, and regulatory dimensions of GBCA toxicity, emphasizing novel concepts like nanoparticle formation.

## Structural classification

GBCAs are primarily categorized by ligand structure: (1) linear GBCAs, which are characterized by flexible, open-chain ligands surrounding the Gd ion and (2) macrocyclic GBCAs, which employ rigid, cage-like ligands providing more secure encapsulation of the Gd ion. Examples of the linear GBCAs include gadodiamide (Omniscan), gadopentetate dimeglumine (Magnevist), and gadobenate dimeglumine (MultiHance). Typically, linear agents have lower stability and are more prone to releasing Gd (Frenzel et al. 2008; Kanal and Tweedle 2015). Examples of the macrocyclic GBCAs are gadoterate meglumine (Dotarem), gadobutrol (Gadavist/Gadovist), and gadoteridol (ProHance).

### Ionic classification

GBCAs are further subdivided by electrical charge. Thus, ionic GBCAs carry a net charge and interact ionically with counterions, while non-ionic GBCAs are electrically neutral. While charge contributes to classification, the main factors influencing in vivo Gd chelate stability and dissociation are ligand structure (linear vs. macrocyclic) and kinetic inertness, more so than just the ionic property (Port et al. 2008; Idée et al. 2009). Non-ionic linear agents might show better tolerability but potentially slightly lower stability than their ionic linear counterparts (Schmitt-Willich 2007).

### Stability parameters

Two key metrics define GBCA stability: (a) thermodynamic stability, quantified by log K(GdL), represents the equilibrium constant for the Gd–ligand binding. Higher values signify stronger binding and increased stability. Log K(cond) denotes stability at physiological pH. Macrocyclic agents generally have higher thermodynamic stability (log K(GdL) ~ 20–25) compared to linear ones (log K(GdL) ~ 16–22) (Caravan et al. 2002; Laurent et al. 2006), and (b) kinetic inertness, measured by the dissociation rate constant (kobs), shows how rapidly the Gd–ligand complex disassembles. Lower values indicate slower dissociation and enhanced in vivo stability, even under demanding biological conditions. Macrocyclic agents typically show much greater kinetic inertness (kobs ~ 10⁻⁷ s⁻^1^) vs. linear agents (kobs ~ 10⁻^4^ s⁻^1^) (Cacheris et al. 1990; Sørensen and Faulkner 2018).

### Clinical classification

For clinical practice, the American College of Radiology (ACR) provides a categorization of GBCAs into three groups based on NSF risk (ACR 2024). Group I (Highest Risk) contains linear agents, such as gadodiamide (non-ionic) and gadopentetate dimeglumine (ionic), group II (Intermediate Risk) encompasses linear ionic agents with some protein binding, such as gadobenate dimeglumine, and group III (Lowest Risk), includes all macrocyclic agents, for example, gadoterate meglumine, gadobutrol, and gadoteridol. This classification system helps guide clinical choices, particularly regarding patients with compromised renal function or those anticipated to undergo multiple contrast examinations (Kanal et al. 2013; Welker et al. 2025).

## Clinical spectrum of gadolinium toxicity

### Nephrogenic systemic fibrosis (NSF)

NSF is the most widely known and severe manifestation of Gd toxicity (Starekova et al. 2024; Welker et al. 2025). First identified in 1997 as “nephrogenic fibrosing dermopathy” and later recognized as systemic, NSF causes fibrosis in skin, joints, and internal organs, primarily affecting patients with severe kidney problems (Cowper et al. 2000; Grobner 2006; Woolen et al. 2020). NSF commonly presents as symmetrical thickening and hardening of the skin, typically initiating in the lower limbs and progressing upwards. Skin might take on a “peau d’orange” texture with discoloration, bumps, and plaques. Joint contractures frequently occur, severely restricting movement. In advanced stages, fibrosis can affect internal organs, such as the heart, lungs, liver, and muscles, leading to higher mortality (Ting et al. 2003; Daram et al. 2005). NSF has been reported almost exclusively in individuals with severe renal impairment (eGFR < 30 mL/min/1.73 m^2^), particularly those on dialysis. Incidence rates peaked in the early 2000 s but decreased sharply after the link with GBCAs was recognized and preventive measures were adopted (Todd et al. 2007; Thomsen 2009).

The typical GBCA dose for MRI is 0.1 mmol/kg body weight, administered intravenously. NSF symptoms typically appear from weeks to months post-exposure, with onset reported from 2 weeks to 3 months in most cases (Marckmann et al. 2006). Regulatory registries (e.g., US FDA, EMA, Health Canada, Japan PMDA) confirm higher NSF risk with linear, non-ionic agents, such as gadodiamide (Omniscan) and gadoversetamide (OptiMARK), leading to restrictions or suspensions (EMA, 2017a; US FDA, 2017a). Macrocyclic agents (e.g., gadoterate meglumine) show minimal to no NSF association at standard doses.

The pathogenesis of NSF is believed to stem from the activation and multiplication of circulating fibrocytes. These cells enter tissues and transform into collagen-producing fibroblasts (Cowper and Bucala 2003). Gadolinium is thought to initiate this cascade through various mechanisms, such as upregulating monocyte chemoattractant protein-1 (MCP-1), transforming growth factor-beta (TGF-β), and possibly NADPH oxidase 4 (Nox4) (Wermuth and Jimenez 2014). More complex mechanisms involving the immune system (e.g., inflammasome activation), mitochondrial damage, and oxidative stress have also been proposed and are discussed further in the context of downstream cellular effects*.* Diminished renal clearance extends GBCA circulation time in patients with kidney impairment, enlarging the window for Gd release, especially from less stable linear agents, thereby facilitating this pathological process (Broome et al. 2007). The connection between GBCAs and NSF is robust, with epidemiological data indicating a dose-dependent risk (Collidge et al. 2007; Kuo et al. 2007). While patient-specific factors such as age or sex may have some influence, the presence of severe renal impairment is the single most critical predisposing factor. Linear, non-ionic agents like gadodiamide present the highest risk. Macrocyclic agents, at standard doses in patients with renal impairment, have not shown a conclusive link to NSF (Thomsen et al. 2013). The inclusion of excess ligand in agents, such as gadodiamide and gadoversetamide, which have been administered in millions of doses worldwide, acts as a concurrent chelating agent, potentially reducing NSF incidence by mitigating free Gd release (Semelka et al. 2019).

The spectrum of GD toxicity spans acute, subacute, and chronic manifestations (Table 1). While NSF represents the most severe acute presentation, emerging evidence highlights long-term deposition-related effects even in patients with normal renal function. In general, the larger the dose of GBCA administration, including multiple administrations, the more severe the disease (this applies to NSF and GDD). However, it should be appreciated this can also arise from administration of a small dose, such as 1 ml of GBCA in MR arthrography. The current theory is that the disease reflects a combination of immunogenicity and toxicity, where the immunologic response allows for the development of a full range of toxic effects of the heavy metal Gadolinium.

**Table 1:** Clinical manifestations of gadolinium toxicity

Entity	Patient population	Temporal association	Major clinical manifestations	Objective findings	Strength of evidence
Nephrogenic systemic fibrosis (NSF)	Primarily patients with severe renal impairment (eGFR < 30 mL/min/1.73m^2^)	Weeks to months after GBCA exposure	• Skin thickening and hardening • “Peau d’orange” appearance • Joint contractures • Pain and pruritus • Possible internal organ fibrosis	• Characteristic histopathology • CD34 + fibrocytes • Increased dermal cell count • Collagen deposition • Gd detection in tissue	Strong • Epidemiological studies • Clear dose–response relationship • Plausible biological mechanism
Brain gadolinium deposition	Patients with normal or impaired renal function receiving multiple GBCA doses	Cumulative over multiple exposures	• Generally asymptomatic • Possible cognitive changes (controversial)	• T1 hyperintensity in dentate nucleus and globus pallidus • Gd detection in brain tissue on autopsy	Moderate • Signal changes well-documented • Tissue Gd confirmed • Clinical significance unclear
Gadolinium deposition disease (GDD)	Patients with normal renal function	Hours to weeks after GBCA exposure	• Persistent headache • Bone/joint pain • Chronic fatigue • Mental fog/confusion • Skin changes • Burning/tingling sensations	• No standardized objective findings • No established biomarkers • Symptom overlap with other conditions	Limited • Primarily case reports/series • No controlled studies • Subjective symptoms • No specific diagnostic test
Acute reactions	General population	Minutes to hours after GBCA exposure	• Nausea, vomiting • Skin rash/hives • Anaphylactoid reactions (rare) • Pain at injection site	• Objective physical findings of hypersensitivity • Vital sign changes in severe cases	Strong • Well-documented adverse events • Clear temporal association • Established incidence rates

### Gadolinium deposition and retention

Beyond NSF, the pharmacokinetics of GBCAs have come under scrutiny as broader anxieties about Gd retention have emerged (McDonald and McDonald 2020). While designed for rapid renal excretion, their in-vivo behavior is complex, influenced by chelate stability, protein binding, and other biological interactions. Since 2014, mounting evidence demonstrates Gd accumulation in diverse tissues, even among patients with normal kidney function, contradicting the prior assumption of complete GBCA clearance (Kanda et al. 2014; McDonald et al. 2015). Regarding tissue localization, deposition happens in the brain, bone, and other tissues. Within the brain, progressive T1 signal hyperintensity within the dentate nucleus and globus pallidus was noted after multiple administrations of primarily linear GBCAs (Ramalho et al. 2015; Jost et al. 2016; Green et al. 2022). Post-mortem analyses verified Gd presence in these regions, correlating with the number of prior GBCA exposures (McDonald et al. 2017). These studies involved patients who died of causes unrelated to GBCA administration, such as cardiovascular events or malignancies, indicating that Gd deposition is not directly linked to mortality but reflects cumulative exposure. Although early studies emphasized linear agents, later research also found Gd in the brain following macrocyclic agent use, albeit usually at lower concentrations (Robert et al. 2015; Behzadi et al. 2018). Bone serves as a major Gd reservoir, showing higher concentrations than other tissues (Darrah et al. 2009; Ramalho et al. 2017). Gd deposition in bone occurs across various types (e.g., long bones and skull), with higher concentrations in cortical bone due to its mineral matrix. This bone deposition can last for years, potentially acting as a long-term source for slow Gd release (Darrah et al. 2009). Gd has also been identified in the skin, liver, and kidneys of individuals after previous GBCA exposure (van der Molen et al. 2024). The pharmacokinetic profile of GBCAs shows rapid distribution to extracellular spaces, with a volume of distribution (Vd) of ~ 0.2–0.3 L/kg. Elimination is primarily renal (half-life ~ 1.5–2 h in normal renal function), but lipophilicity and protein binding (e.g., gadobenate’s 10–15% protein binding) influence tissue distribution. Linear agents, with lower stability (log K ~ 16–22), are more prone to deposition than macrocyclic agents (log K ~ 20–25) due to differences in dissociation rates (Frenzel et al. 2008).

Animal studies revealed wider distribution across various organ systems (Robert et al. 2015, 2018), while Le Fur et al. (2023) demonstrated in rats that Gd from both linear and macrocyclic GBCAs distributed to multiple tissues, including brain, bone, and kidneys, with varying chemical speciation. These findings suggest that Gd may persist as intact chelates, free ions, or precipitated forms, highlighting the complexity of long-term retention mechanisms (Le Fur et al. 2023).

Although most GBCA is eliminated within days by individuals with normal kidney function, trace Gd levels remain detectable in urine months or years later, suggesting slow release from tissue reservoirs (Pietsch et al. 2009; Kanda et al. 2015a,b). The clinical relevance of Gd deposition, particularly in the brain, is not yet fully established. While some studies hint at possible links to subtle neurological problems like cognitive shifts or fatigue, causality remains unproven. Most research has not shown overt neurological issues directly caused by brain Gd deposition, although subtle effects, particularly from repeated exposure, cannot be definitively excluded (Welk et al. 2016; Forslin et al. 2017). Regarding this, a recent study utilizing the Korean National Health Insurance Service Database has reported an association between GBCA exposure and Parkinson’s disease, with no such association in patients who did not receive GBCAs, suggesting a potential neurological risk that warrants further investigation (Kim et al. 2025). Gulani et al. (2017) provided consensus guidelines, recommending judicious GBCA use while noting the limited evidence of clinical harm from brain deposition. In turn, Choi and Moon (2019) reviewed deposition pathways and patterns, highlighting differences between linear and macrocyclic agent types.

### Gadolinium deposition disease (GDD)

Some patients experience persistent symptoms following GBCA administration, leading to the proposed diagnosis termed Gadolinium Deposition Disease (GDD) (Harvey et al. 2020; Qu et al. 2024). GDD is a condition involving persistent symptoms following exposure to GBCA. The disease often arises within 24 h but may arise up to a month following the GBCA administration. Common symptoms include cognitive impairment, bone pain (distinctive is rib pain), skin pain, muscle fasciculations, and pins and needles sensation in the fingers (Semelka and Ramalho 2023). Patient advocacy groups have surfaced, increasing awareness and urging further investigation. Establishing causality and precise diagnostic criteria remains problematic (Burke et al. 2016; Semelka et al. 2016a, b). The term GDD, while proposed by researchers with extensive experience in Gd-related effects, is supported by clinical observations spanning several years, though its diagnostic criteria are still under refinement (Semelka et al. 2016a). Critics note symptom similarities with conditions, such as fibromyalgia and chronic fatigue syndrome, while some researchers suggest GDD may represent a specific subtype of these conditions, sharing mechanistic overlaps (Semelka et al. 2016b). Reported symptoms cover persistent headaches, bone/joint discomfort, chronic fatigue, mental fog, skin alterations (thickening, rash), burning/tingling sensations, and sensory disturbances. Parillo et al. (2023) reviewed skin deposition and toxicity in patients whose renal function was normal, suggesting a possible mechanistic link to Gd exposure. However, objective diagnostic markers for GDD are lacking (Semelka et al. 2016a,b). Symptom overlap with fibromyalgia and chronic fatigue syndrome complicates diagnosis. The temporal connection to GBCA use forms the primary basis for suspicion (Ramalho et al. 2016). While Gd deposition is confirmed, its direct causal role in these reported symptoms is not definitively proven. Nonetheless, from the patient’s view, the temporal association between receiving a GBCA and symptom onset is often compelling, motivating the search for answers and therapies. Suggested potential mechanisms include immune responses, mitochondrial damage, and direct cellular injury from free Gd or nanoparticles (Wermuth and Jimenez 2012; Do et al. 2020). Recent work by Maecker et al. (2021, 2022) at Stanford’s Human Immune Monitoring Center has explored cytokine profiles in GDD patients undergoing chelation therapy, identifying potential immune-mediated mechanisms that could contribute to symptomology, underscoring the need for further immunological studies. GDD research remains in early stages, relying mainly on case reports/series (Burke et al. 2016; Semelka and Ramalho 2021). Controlled studies are necessary to better define this condition and establish diagnostic criteria. Lyapustina et al. (2019) pointed out evaluation difficulties, stressing the need to exclude other conditions due to non-specific symptoms and the lack of validated GDD biomarkers. Semelka et al. (2016a,b) proposed diagnostic criteria, indicating symptom onset within hours to a month post-GBCA, with a cluster, including central torso pain, neuropathy, headache, and cognitive issues.

### Other potential toxicities

Rare occurrences of Acute Kidney Injury (AKI) after GBCA administration have been noted, though the frequency has been substantially lower than with iodinated contrast agents (Kalb et al. 2008; Bhaskaran et al. 2010). Furthermore, local problems from contrast extravasation are a consideration. Granata et al. (2016) reviewed contrast media extravasation, observing that while usually mild, severe instances needing surgery can happen, highlighting correct injection protocols. Regarding hypersensitivity reactions, immediate reactions occurred in ~ 0.01–0.3% of cases, with severe anaphylactoid events being very uncommon (0.001–0.01%) (Dillman et al. 2007; Granata et al. 2016). Management of these acute events follows standard protocols for allergic-like reactions, including the use of antihistamines or corticosteroids, with emergency measures for severe cases. Neurotoxicity has also been documented with accidental intrathecal injection or significant blood–brain barrier compromise, manifesting as confusion, drowsiness, visual problems, and seizures (Ray et al. 1998; Hui and Mullins 2009; Bower et al. 2019).

## Mechanisms of gadolinium release, deposition, and toxicity

Understanding how Gd detaches from chelates, deposits in tissues, and causes toxicity is key for creating safer agents and reducing risks. Some findings have suggested mechanisms are more complex than initially believed (Coimbra et al. 2024).

### Traditional view

Transmetallation involves Gd^3^⁺ exchange with endogenous metals (such as Zn^2^⁺, Cu^2^⁺, Fe^3^⁺, and Ca^2^⁺), releasing free, toxic Gd^3^⁺ (Tweedle 1992; Idée et al. 2006). The relative stability of metal–ligand complexes influences the likelihood of this exchange (Wedeking et al. 1989). Key determinants include GBCA stability (linear agents are more susceptible than macrocyclics) (Laurent et al. 2001), exposure duration (prolonged with renal impairment) (Kanda et al. 2015b), concentration of competing metals (Wright et al. 2014), and the biological milieu (pH, protein binding) (Cao et al. 2016). However, transmetallation alone fails to fully explain all observed Gd deposition patterns, particularly the detection of Gd within the brain following administration of highly stable macrocyclic agents (Radbruch et al. 2016; Splendiani et al. 2018; Cowling and Frey 2019).

### Role of acidic environments

Acidic conditions markedly influence Gd release, potentially explaining deposition in specific cellular compartments (Le Fur and Caravan 2019). GBCA stability generally lessens at lower pH, with linear agents being especially vulnerable to acid-driven dissociation. Macrocyclic agents usually maintain better stability under acidic conditions (Aime et al. 1998; Uzal-Varela et al. 2022).

### Precipitation/nanoparticle pathway

**Fig. 1:**
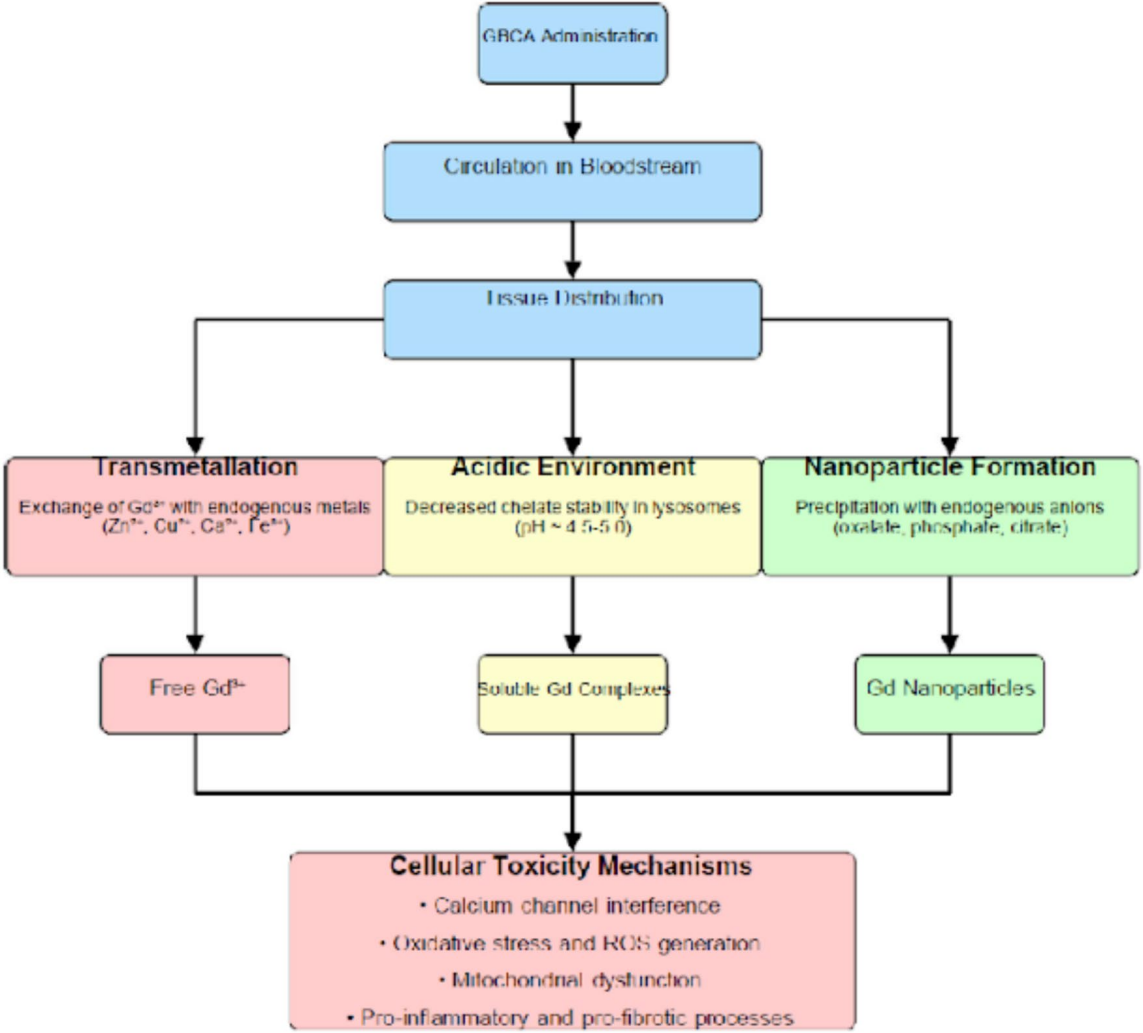
Proposed mechanisms of gadolinium release and toxicity

Figure 1 provides a visual summary of the proposed mechanisms by which Gd is released from contrast agents and subsequently exerts toxic effects, including the roles of transmetallation, acidic dissociation in lysosomes, and nanoparticle formation. Some studies have suggested an alternative pathway: the generation of insoluble Gd-containing nanoparticles. This might occur with both linear and macrocyclic agents (Gianolio et al. 2017; Taupitz et al. 2013). A recent investigation by Henderson et al. (2025) provided strong experimental support for this mechanism. These authors showed that both linear and macrocyclic GBCAs can dechelate and subsequently precipitate as gadolinium oxalate in acidic, lysosome-like environments. That in vitro study confirms even macrocyclics like Dotarem can be susceptible to oxalate-induced precipitation, especially when proteins are present and pH is low. It supports the biological feasibility of nanoparticle formation contributing to Gd retention and toxicity. However, the hypothesis regarding oxalate-driven dechelation and nanoparticle formation requires validation by independent research groups, such as those led by Dr. Pietsch at Bayer or the Guerbet research team, to confirm its relevance in vivo (Semelka et al. 2023). The process yields gadolinium oxalate precipitates, potentially serving as precursors to observed intracellular nanoparticles. The body’s environment actively affects Gd dechelation and precipitation. Proteins like bovine serum albumin (BSA) have demonstrated an ability to accelerate dechelation, suggesting biological molecules actively participate (Lux et al. 2015). The complex chemistry, involving ligand design and metal coordination, impacts stability and dechelation potential (Wahsner et al. 2019). Besides oxalate, other endogenous anions such as phosphate and citrate can also promote Gd precipitation and nanoparticle formation, highlighting intricate in vivo interactions (Yang and Chuang 2012; García et al. 2017; Marasini et al. 2021). Until validated, caution has been advised against recommending dietary restrictions, such as avoiding oxalate-rich foods, as oxalates may be incidental rather than causative in Gd retention. This mechanism offers a plausible rationale for Gd deposition beyond just transmetallation, covering observations with both linear and macrocyclic types. It implies even highly stable macrocyclics might dechelate under specific biological conditions (Aime and Caravan 2009). Frenzel et al. (2017) measured residual Gd in the brain after repeated GBCA administrations, finding a significant amount present in a soluble, but not necessarily fully chelated form, further supporting complex retention mechanisms. Emerging data suggest Gd-containing nanoparticles could initiate neuroinflammatory or fibrotic processes, acting either as inert storage or as active toxic agents via interactions with cells and organelles (Henderson et al. 2025). Whether these nanoparticles are biologically inactive or harmful remains under investigation. While transmetallation was historically considered the primary pathway for Gd release, recent evidence demonstrates that nanoparticle formation via endogenous ligands (e.g., oxalate in lysosomal environments) may represent a parallel mechanism—even for macrocyclic agents (Rogosnitzky and Branch 2016; Coimbra et al. 2024). This challenges the assumption that kinetic inertness alone ensures safety and underscores the need for agent-specific risk assessments. To address toxicity concerns, alternative approaches like Gd-grafted nanodiamonds, which are coated with PVP to prevent Gd^3^⁺ release, have shown promise. These nanoparticles exhibit high relaxivities (*r*_1_ = 33.4 mM⁻^1^ s⁻^1^, *r*_2_ = 332 mM⁻^1^ s⁻^1^) compared to Dotarem (*r*_1_ = 3.6 mM⁻^1^ s⁻^1^, *r*_2_ = 4.3 mM⁻^1^ s⁻^1^) and remain stable for years, potentially offering a safer MRI contrast agent (Panich et al. 2016, 2019, 2021; Chizhikova et al. 2024).

### Downstream cellular effects

Once Gd is released (as free Gd^3^⁺ or within nanoparticles), several toxic pathways can be activated: (1) free Gd^3^⁺, owing to its ionic radius similarity to Ca^2^⁺, can disrupt voltage-gated calcium channels and calcium-dependent enzymes, impairing cellular functions (Lansman 1990; Idée et al. 2006); (2) inflammation arises when Gd deposits provoke local inflammatory reactions, including macrophage activation and cytokine release, contributing to tissue damage and fibrosis (Vakil et al. 2009; Edward et al. 2010); (3) Gd can also promote the generation of reactive oxygen species (ROS), inflicting oxidative damage on proteins, lipids, and DNA (Niendorf et al. 1991; Stojanov et al. 2016a, b); (4) evidence indicates Gd can impede mitochondrial function, affecting energy production and potentially triggering apoptosis (Spencer et al. 1997); (5) in vitro studies as that conducted by Erdoğan et al. (2021), revealed dose-dependent GBCA toxicity on neuronal cells, with linear agents causing more damage than macrocyclics; (6) in NSF, Gd appears to stimulate fibroblast growth and collagen synthesis through upregulation of profibrotic cytokines and growth factors like TGF-β (Sieber et al. 2008a, b; Gou et al. 2010); and (7) Gd-containing nanoparticles might exert biological effects distinct from free Gd^3^⁺, interacting with cell membranes, proteins, or organelles, or acting as a reservoir for gradual Gd release (De León-Rodríguez et al. 2009; Coimbra et al. 2024).

## Risk factors for gadolinium toxicity/retention

Identifying factors elevating susceptibility to Gd toxicity assists in risk assessment and prevention.

### Renal function

Compromised renal function is the most critical risk factor for Gd toxicity, especially NSF. Risk inversely correlates with eGFR; the highest risk is in patients with eGFR < 30 mL/min/1.73 m^2^, particularly those on dialysis or with AKI (Collidge et al. 2007; Reilly 2008). Reduced renal clearance prolongs GBCA circulation, increasing opportunities for Gd release via transmetallation or other pathways (Sieber et al. 2008a, b). Standard eGFR calculations might not always accurately reflect true GFR, particularly in individuals with unusual body size, critical illness, or fluctuating renal status, potentially leading to flawed risk assessment (Stevens et al. 2009; Rule and Glassock 2013).

### GBCA type and stability

The chemical structure and stability of GBCAs heavily impact toxicity risk. Thus, linear agents, especially non-ionic types like gadodiamide, pose a substantially higher NSF risk than macrocyclic agents (Cowling and Fray 2019; Semelka et al. 2019). Linear agents also exhibit greater tissue deposition, though all classes contribute somewhat (Runge 2016). Among linear agents, ionic ones generally possess better stability than non-ionic ones, potentially implying lower risk (Morcos 2008). The American College of Radiology’s three-group classification has provided a practical guide for agent selection based on risk (ACR 2024). A meta-analysis by Woolen et al. (2020) supported this, finding a very low (possibly zero) NSF risk with Group II agents even in patients with stage 4/5 CKD, unlike the higher risk with Group I agents.

### Cumulative dose

Data consistently demonstrate a dose-dependent link for both NSF risk and tissue deposition. Repeated GBCA administrations increase cumulative Gd burden. Studies connect the number of prior administrations to the extent of brain signal alterations or tissue Gd levels (Gulani et al. 2017; Radbruch et al. 2017a, b, c). The interval between administrations might also influence risk, but optimal timing was unclear (Errante et al. 2014).

### Other potential risk factors

Evidence for other factors modifying Gd toxicity risk is less definitive. Concurrent inflammation might enhance Gd release and tissue injury through increased vascular permeability and acidic conditions (Bhave et al. 2008; Perez-Rodriguez et al. 2009; Wahsner et al. 2019). Gadolinium also crosses the placenta, causing fetal deposition. Although teratogenicity is not confirmed, caution is advised (Webb et al. 2005; De Santis et al. 2007; Kong et al. 2025). Moreover, conditions such as multiple sclerosis, tumors, or inflammation disrupting the BBB can facilitate Gd entry into brain parenchyma (Miller et al. 2015; Roberts et al. 2016). Furthermore, children might be more vulnerable due to developing organs, maturing BBB, and a longer potential lifespan for effects (Flood et al. 2017; Lohrke et al. 2017). In addition, individual genetic variations in metal handling or inflammatory responses could affect susceptibility, but specific markers are yet to be identified (Golding et al. 2008; de Frutos et al. 2023).

## Diagnosis and monitoring

Reliable diagnosis and monitoring for Gd-related toxicities, particularly beyond NSF, remain challenging.

### Clinical assessment

For NSF, diagnosis combines characteristic clinical signs (skin thickening, contractures) with histopathology (increased dermal cells, CD34 + fibrocytes, collagen) within the context of GBCA exposure and renal dysfunction (Cowper 2008; Deng et al. 2010). For GDD, no standardized diagnostic criteria exist. Assessment involves documenting symptom timing relative to GBCA use, excluding other causes, and potentially confirming Gd retention (Ramalho et al. 2017; Parillo et al. 2023).

### Imaging assessment

Progressive T1 hyperintensity noted in the dentate nucleus and globus pallidus on unenhanced MRI acts as a radiological sign of brain Gd deposition, mainly linked to linear agents (Robert et al. 2015; Stojanov et al. 2016a, b; Quattrocchi et al. 2019). These signal changes do not perfectly align with Gd concentration and might miss deposition below detection limits. The link between signal changes and clinical symptoms remains uncertain (Kang et al. 2018).

### Laboratory assessment

Definitive proof of Gd deposition needs tissue sampling, typically restricted to research or post-mortem studies due to invasiveness (Xia et al. 2010). Urine/blood Gd measurements confirm recent exposure but reflect clearance or mobilization, not total body burden or tissue levels. Normal elimination kinetics complicate interpretation, as Gd might be detectable for days/weeks even without abnormal retention (Huckle et al. 2016; Fraum et al. 2017). Currently, no validated biomarkers exist for Gd toxicity or problematic retention, hindering early detection and prognosis (Rasschaert et al. 2018).

### Monitoring challenges

The potential for delayed symptom onset and the uncertain clinical meaning of Gd deposition complicate long-term monitoring (Semelka et al. 2016a, b). Monitoring approaches must balance surveillance needs with resource use and potential patient anxiety arising from uncertain findings (Tibussek et al. 2017). The lack of clear clinical correlation for findings like brain hyperintensity can cause significant worry for patients undergoing monitoring.

## Management and mitigation

Managing Gd-related risks involves prevention, careful agent choice, and weighing benefits against risks (Scarciglia et al. 2025).

### Risk stratification and informed consent

Screening for risk factors (renal impairment, inflammation, prior reactions) should inform decisions (Shellock and Spinazzi 2008). Patients need information about potential risks, including Gd retention, tailored to their individual factors and the selected agent (ACR 2024). Effective risk communication is crucial. This requires explaining not only established risks like NSF (in susceptible patients), but also uncertainties surrounding Gd deposition and GDD, ensuring patients can make truly informed choices collaboratively with their clinicians. Openly addressing patient concerns and questions is paramount.

### Agent selection and dose optimization

Balancing diagnostic need with safety is essential, especially in high-risk individuals (Fretellier et al. 2011; Quattrocchi et al. 2024). Macrocyclic agents are generally preferred due to higher stability and lower deposition, particularly for patients with risk factors or needing repeat scans (Layne et al. 2018). Specific guidance exists for high-risk groups like those with chronic kidney disease (CKD), reinforcing risk stratification by agent class and renal function (Rudnick et al. 2021). Group I agents should be avoided in patients with severe renal impairment (eGFR < 30 mL/min/1.73 m^2^) and used very cautiously, if ever, in those requiring multiple scans (ACR 2024). Employing the lowest effective diagnostic dose minimizes total Gd exposure and risks (ESUR 2018; Islam and Tsnobiladze 2024), while careful planning can prevent unnecessary repeat scans, especially at short intervals (Wang et al. 2011). For high-risk patients, consider non-contrast MRI or alternative imaging methods if suitable (Tong et al. 2014; Runge 2017). Current risk mitigation strategies emphasize agent selection, dose optimization, and patient screening (Table 2). These measures are particularly critical in high-risk populations, such as those requiring repeated GBCA exposure.

**Table 2:** Risk management strategies for GBCA use

Patient risk category	Risk assessment	Agent selection	Dose considerations	Monitoring recommendations	Alternative approaches
Severe renal impairment (eGFR < 30 mL/min/1.73m^2^)	• Measure eGFR prior to GBCA • Assess hydration status • Review prior GBCA exposure	• Macrocyclic agents only (Group III) • Avoid linear agents (Group I/II)	• Minimum effective dose • Avoid repeat injections • Minimum 7-day interval between doses	• Document GBCA type and dose • Clinical follow-up for NSF symptoms • Consider dermatology evaluation if skin changes	• Non-contrast MRI protocols • Alternative imaging modalities • Ultrasound or CT when appropriate
Moderate renal impairment (eGFR 30–60 mL/min/1.73m^2^)	• Measure eGFR prior to GBCA • Review prior GBCA exposure • Consider risk factors	• Preferably macrocyclic agents (Group III) • May use Group II with caution • Avoid Group I	• Standard dose • Minimize repeat injections • At least 48-h interval between doses	• Document GBCA type and dose • Routine clinical follow-up	• Consider non-contrast MRI if diagnostically adequate • Lower dose protocols
Normal renal function with multiple exposures	• Review prior GBCA exposure • Estimate lifetime cumulative dose	• Preferably macrocyclic agents (Group III) • May use Group II • Consider Group I only if specific indication	• Standard dose • Minimize unnecessary repeat scans	• Document GBCA type and dose • Consider baseline MRI for future comparison	• Optimize protocols to reduce need for repeat scans • Consider alternative sequences
Pediatric patients	• Assess renal function • Consider developmental factors • Evaluate long-term risk	• Macrocyclic agents preferred (Group III)	• Weight-based dosing • Minimum effective dose	• Document GBCA type and dose • Long-term follow-up consideration	• Non-contrast protocols when possible • Alternative imaging modalities
Pregnant/breastfeeding	• Assess benefit vs. risk to mother and fetus/infant • Consider gestational age	• Macrocyclic agents if GBCA necessary	• Minimum effective dose	• Document GBCA type and dose • No specific monitoring required for breastfeeding	

The standard route of administration for GBCAs is intravenous (IV). The term ‘minimum effective dose’ refers to the lowest diagnostically adequate dose required for the clinical indication. This is often the standard approved dose (e.g., 0.1 mmol/kg body weight) but should always be determined on a case-by-case basis to balance diagnostic yield and potential risks. Doses for pediatric patients are weight-based

### Management of established toxicity

For NSF management, primarily supportive care focusing on physical therapy, skin treatments, and optimizing renal function (including transplantation) is recommended (Swaminathan and Shah 2007). No definitive cure exists; therefore, management relies on symptom control. Various treatments (anti-inflammatories, chelation) have been tried with inconsistent outcomes (Semelka et al. 2018).

### Chelation therapy

Chelation therapy is a well-established approach for treating heavy metal poisoning, utilizing agents, such as ethylenediaminetetraacetic acid (EDTA), dietheylene triamine pentaacetic acid (DTPA), 2,3-dimercapto-1-propanol (BAL), and D-penicillamine (D-PA) since the 1950 s, with more recent agents, including dimercaptosuccinic acid (DMSA), 2,3-dimercaptopropane-1-sulfonate (DMPS), and Tiron. Typical routes and doses of administration for GBCA toxicity are the following. DTPA is intravenously (1 g/day), administered as CaDTPA), with limited data for its use in GBCA toxicity, given at single or multiple doses, aiming to enhance urinary excretion of Gd. EDTA is usually given as CaNa_2_EDTA, being administered in adults at 1000 mg/m^2^ (IV or IM) weekly, although its affinity for Gd is lower than that of DTPA. On the other hand, DMSA is usually administered orally at doses of 10 mg/kg/day every 8 h, for 5 days, but Gd chelation efficacy is uncertain, and not preferred for acute toxicity.

In general, chelating agents effectively counteract heavy metal toxicity but can also cause adverse effects and deficiencies in essential elements, often necessitating mineral supplementation (Domingo 1989, 1998, 2006). Recent research has also explored bioactive compounds with antioxidant and anti-inflammatory properties for chelation, alongside the development of orally administrable chelators suitable for home health care. Balali-Mood et al. (2025) reviewed current antidotes for metal poisoning, highlighting DMSA and DMPS as safe oral chelators for various metal toxicities, which may have relevance for Gd.

In the context of Gd, chelation therapy for this element removal remains controversial and is primarily used off-label (Maecker et al. 2021). Layne et al. (2018) reviewed the topic, concluding that there is insufficient evidence to define Gadolinium Deposition Disease as a distinct condition and cautioning against chelation therapy due to unproven effectiveness and potential risks. This team, however, are scientists and not physicians, so would have limited direct clinical experience. Very few controlled studies validate the efficacy or safety of chelation for Gd, with most data derived from case reports or series (Semelka et al. 2018, 2022; Maecker et al. 2021, 2022). Semelka and Ramalho (2023) suggested that diethylene triamine penta-acetic acid (DTPA) was the most effective chelating agent for Gd due to its high affinity, proposing its use to mitigate GDD. DTPA’s log stability constant with Gd (~ 22) is significantly higher than that of EDTA (~ 17), indicating a 300,000-fold greater binding strength, making DTPA a more suitable chelator. EDTA, evaluated in early GBCA development, was deemed too weak for clinical use (Idée et al. 2006). Thus, suggesting EDTA for Gd chelation may be inappropriate due to its lower efficacy and potential risks. Animal studies suggest chelation reduces Gd burden (Rees et al. 2018; Sun et al. 2022), with DTPA decreasing bone retention by 40% in rats (Rogosnitzky and Branch 2016), but human data remain rather limited. In addition, hydroxypyridinone-based ligands (HOPO), such as Me-3,2-HOPO, have shown promise in mitigating Gd deposition in animal models, offering another potential chelating agent for future exploration (Sun et al. 2022). Risks of hypocalcemia, nephrotoxicity, and essential metal depletion necessitate caution until controlled trials validate protocols (Cunningham et al. 2024), while Henderson et al. (2025) advised against chelation without stronger evidence, citing the lack of robust data on its benefits for Gd retention. However, chelation remains the only currently effective treatment for GDD, mirroring its role in other heavy metal toxicities, and should not be dismissed without further investigation (Semelka and Ramalho 2023). Recently, Schilling et al. (2025) assessed in volunteers the efficacy of EDTA in mobilizing toxic metals, including lead, cadmium, and Gd while minimizing the loss of essential elements, such as Mn and Cu. Gd excretion increased by up to 78,000% even at 0.5 g. This finding would highlight the potential use of EDTA to reduce long-term Gd burden post-MRI. Nevertheless, given DTPA’s superior stability, it remains the preferred agent pending further studies. Controlled clinical trials are essential to determine the optimal chelating agents, timing, dosage, and patient selection for Gd-related toxicities, building on the general chelation principles outlined in earlier studies.

For NSF, no chelation therapy has shown consistent efficacy. Thus, treatment focuses on supportive care, including physical therapy and renal function optimization (Swaminathan and Shah 2007). For GDD, chelation with DTPA has been proposed, showing symptom improvement in series reports, though randomized controlled trials are lacking (Semelka et al. 2018). EDTA, despite recent evidence of mobilizing Gd (Schilling et al. 2025), is less effective due to lower binding affinity. Adverse events, such as hypocalcemia or nephrotoxicity, may require specific monitoring.

## Regulatory perspectives

Global regulatory bodies have addressed emerging Gd safety evidence, balancing diagnostic utility and potential harm. The US FDA implemented several actions: issued a boxed warning in 2007 for NSF risk; added class warnings for Gd retention in 2017; recommended restricted use of specific linear agents, and mandated distribution of medication guides to inform patients (US FDA 2017a, b). The US FDA focused on risk mitigation like medication guides for all GBCA classes, permitting continued use of linear agents with precautions. In turn, the European Medicines Agency (EMA) enacted more restrictive measures (EMA 2017a, b): suspended marketing for four linear GBCAs in 2017 (gadodiamide, gadopentetate dimeglumine, gadoversetamide, gadobenic acid); restricted gadobenic acid to liver imaging; and maintained approval for macrocyclics and liver-specific gadoxetic acid. This divergence highlights challenges regulators face balancing established benefits against emerging, sometimes uncertain, risks. Practice patterns and GBCA availability consequently vary significantly across regions. Some nations follow EMA’s restrictions, others align with the US FDA, while some, like Japan, maintain linear agent approval with specific warnings (Endrikat et al. 2018; Japanese Joint Committee of NSF and Use of Gadolinium Based Contrast Agents 2025). Other regulatory bodies, such as Health Canada or Australia’s Therapeutic Goods Administration (TGA), have also issued communications and restrictions, often aligning closely with either the US FDA or EMA approach depending on their assessment. Regulatory actions have markedly impacted clinical practice, favoring macrocyclics, improving screening, emphasizing benefit–risk assessment, and enhancing patient communication (Gulani et al. 2017; Ramalho et al. 2017; Runge 2018).

## Conclusions and future directions

Gadolinium toxicity ranges from the established NSF entity to the increasingly acknowledged issue of widespread tissue deposition, whose clinical relevance is debated. Emerging mechanistic understanding points to complex processes beyond simple transmetallation, potentially involving Gd-containing nanoparticle formation via interactions with endogenous molecules in specific microenvironments (Taupitz et al. 2013; Gianolio et al. 2017; Marasini et al. 2020; Henderson et al. 2025).

Key implications of this expanded view include: (a) even highly stable macrocyclic GBCAs might dechelate under certain biological circumstances (Aime and Caravan 2009; Iyad et al. 2023), (b) the biological environment plays an active role in Gd release, not just a passive one (Lux et al. 2015), (c) nanoparticle formation could represent a distinct toxicity pathway beyond free Gd^3^⁺ effects (De León-Rodríguez et al. 2009; Rahmani et al. 2024; Afriani et al. 2025). Despite progress, critical knowledge gaps persist. These include: a) the long-term clinical impact of brain and tissue deposition (McDonald et al. 2017), (b) validating “Gadolinium Deposition Disease” as a specific clinical condition (Semelka et al. 2023), (c) the need for reliable biomarkers for Gd toxicity or problematic retention (Choi and Moon 2019), (d) effective treatments for symptomatic Gd retention (Ramalho et al. 2017), and (e) understanding individual susceptibility and risk prediction (Quattrocchi and van der Molen 2017).

Future research priorities should involve longitudinal studies linking Gd deposition to histopathology, developing non-gadolinium alternatives (e.g., iron oxide nanoparticles or Gd-grafted nanodiamonds with PVP coating for enhanced safety), validating biomarkers for early retention detection, and conducting controlled trials on chelation therapy efficacy (Radbruch et al. 2017a; Robert et al. 2018; Panich et al. 2021; Al-Muhanna 2022).

Recent research on non-gadolinium alternatives includes Mn-PyC3A, a manganese-based contrast agent evaluated using PET–MRI in rat models. Zhou et al. (2021) demonstrated that Mn-PyC3A is efficiently eliminated via mixed renal and hepatobiliary pathways, even in renal impairment, with significantly lower retention compared to gadoterate (Gd-DOTA) after 7 days. This suggests Mn-PyC3A could offer a safer profile for MRI contrast, particularly in patients with compromised renal function.

Until these gaps are filled, a cautious approach remains necessary: judicious GBCA use (reserving for clinical need) (ACR 2024), preferring macrocyclics (especially in high-risk patients or those needing multiple scans) (Reiter et al. 2012), considering cumulative dose (Mallio et al. 2020), thorough documentation of GBCA administration to facilitate long-term monitoring (Layne et al. 2018), and open patient communication about risks and uncertainties (Kanal and Tweedle 2015). Engaging patients in shared decision-making, supported by clear and balanced information, will remain essential as understanding evolves.

### Balancing benefits and risks

GBCAs are indispensable diagnostic tools that have significantly advanced medical imaging and patient care. The ongoing task is to balance their clear clinical advantages against potential long-term risks. It is crucial to remember that for many patients, the diagnostic information gained from a GBCA-enhanced MRI significantly outweighs the currently known potential risks, especially when using more stable agents and adhering to screening guidelines. For example, accurate tumor staging, assessment of treatment response in oncology, or identification of inflammatory lesions in multiple sclerosis often relies heavily on GBCA enhancement. The potential harm of a missed or delayed diagnosis must be carefully weighed against the risks tied to Gd exposure. This necessitates refining risk stratification methods (Do et al. 2020), developing patient-specific protocols (Zheng et al. 2022), adapting practices as new data become available (Runge 2017), and ensuring transparent communication among healthcare professionals and patients (Weinreb et al. 2020). Recent insights into Gd precipitation and nanoparticle formation highlight the intricate nature of GBCA–biological system interactions and emphasize the need for continued research to optimize the safety of these valuable diagnostic agents (Kang and Zhao 2022; Iyad et al. 2023; Kawassaki et al. 2023; Maimouni et al. 2025).

## Limitations of current knowledge and this review

Although the present review synthesizes a broad range of literature on Gd toxicity, several limitations should be acknowledged, both within the current body of knowledge and in the scope of this review. There are gaps in evidence. For example, definitive understanding of the long-term clinical significance of Gd deposition, particularly in the brain with normal renal function, remains elusive. Robust longitudinal studies correlating deposition levels with specific clinical outcomes are still needed. Moreover, the existence and diagnostic criteria for ‘Gadolinium Deposition Disease’ (GDD) require further refinement and broader acceptance within the medical community. Much of the evidence relies on case reports and series, often subject to selection bias, making causality difficult to establish. There is also a lack of validated, accessible biomarkers to reliably quantify Gd body burden or identify individuals experiencing Gd-related toxicity beyond NSF. While this review synthesizes preclinical and clinical data, the lack of standardized Gd speciation methods in human tissues limits mechanistic certainty. Moreover, the novel hypothesis of oxalate-driven nanoparticle formation needs independent validation to confirm its clinical relevance. In addition, heterogeneity in GBCA dosing protocols across studies complicates cumulative risk assessments. Regarding mechanistic uncertainty, while transmetallation and nanoparticle formation offer plausible mechanisms, the precise in vivo processes, their relative contributions, and the exact molecular triggers under various physiological conditions require further elucidation.

It should also be noted that this review primarily focused on English-language publications identified through Scopus, PubMed, and Embase up to April 2025. Relevant studies in other languages or additional databases may have been missed. Furthermore, the rapid evolution of this field means new findings may emerge after this review’s completion. In addition, studies often vary significantly in methodology, patient populations, GBCA types used, and outcome measures, making direct comparisons and meta-analyses challenging.

## Data Availability

Data are available from the authors on request.

## Abbreviations

ACR: American College of Radiology
AKI: Acute kidney injury
BBB: Blood–brain barrier
BSA: Bovine serum albumin
CKD: Chronic kidney disease
DTPA: Diethylene triamine penta-acetic acid
EDTA: Ethylenediaminetetraacetic acid
eGFR: Estimated glomerular filtration rate
EMA: European Medicines Agency
FDA: Food and Drug Administration
GBCA: Gadolinium-Based Contrast Agent
Gd: Gadolinium
GDD: Gadolinium deposition disease
HOPO: Hydroxypyridinone
MCP-1: Monocyte chemoattractant protein-1
MRI: Magnetic resonance imaging
NSF: Nephrogenic systemic fibrosis
PVP: Polyvinylpyrrolidone
ROS: Reactive oxygen species
TGF-β: Transforming growth factor-Beta
